# Volume-outcome relationship in corrective surgery for Hirschsprung’s disease: a systematic literature review of direct evidence and an overview of indirect evidence

**DOI:** 10.1007/s00383-025-06117-4

**Published:** 2025-07-21

**Authors:** Valeria Solari, Thomas Boemers, Eberhard Schmiedeke, Wolfram Trudo Knoefel, Michael Böttcher, Ekkehart Jenetzky, Miriam Wilms

**Affiliations:** 1 Department of Paediatric Surgery, Johannes Kepler University, Kepler University Hospital, Linz, Austria; 2 Clinic for Paediatric and Adolescent Surgery and Urology, Children’s Hospital Cologne, Cologne, Germany; 3 Department of Paediatric Surgery and Urology, Centre for Child and Youth Health, Klinikum Bremen-Mitte, Bremen, Germany; 4 Department of General, Visceral, Thorax and Paediatric Surgery, University Hospital Düsseldorf, Düsseldorf, Germany; 5 Department of Paediatric Surgery, University Hospital Mannheim, Heidelberg University, Mannheim, Germany; 6 Faculty of Health/School of Medicine, Witten/Herdecke University, Witten, Germany; 7 Department of Paediatric and Youth Psychiatry and Psychotherapy, Johannes Gutenberg University Mainz, Mainz, Germany; 8 Patient Organization for People With Anorectal Malformations and Morbus Hirschsprung (SoMA E.V.), Munich, Germany

**Keywords:** Hirschsprung’s disease, Volume-outcome relationship, Indirect evidence, GRADE

## Abstract

Hirschsprung’s disease (HD) is a rare congenital condition requiring complex corrective surgery. We reviewed available direct evidence on the volume–outcome relationship for HD corrective surgery and assessed the applicability of indirect evidence based on GRADE (Grading of Recommendations Assessment, Development, and Evaluation) recommendations for rare diseases. Three retrospective cohort studies analysing the volume-outcome relationship for HD surgery met the inclusion criteria. In all studies, the high-volume threshold was below 12 HD corrective surgeries per year. No significant volume–outcome relationship was found for outcomes such as readmission or intestinal perforation. Faecal incontinence was not assessed. No risk-adjustment was performed. We applied the GRADE framework and explored indirect evidence from adult colorectal surgery, which shares technical similarities and complexity with HD corrective procedures. Multiple studies in adult colorectal surgery demonstrate a clear volume–outcome relationship, with improved outcomes mostly observed in centres performing more than 20 rectal resections annually. Direct evidence for a volume–outcome relationship in HD surgery cannot be established or refuted due to low caseloads and decentralization. Indirect evidence from adult colorectal surgery with comparable case complexity and the same core outcome parameters suggests the presence of a volume-outcome relationship in HD corrective surgery.

## Introduction

Hirschsprung’s disease (HD) is a rare congenital anomaly, with an estimated incidence of 1.3 per 10,000 live births [1]. Corrective surgery is the mainstay of treatment, with the primary objective being the removal of the aganglionic segment with the preservation of the anal canal and sphincter mechanism to support regular bowel movements and maintain faecal continence. As the rectum is always at least partially aganglionic, surgery includes a rectal resection with a low rectal anastomosis. Surgical correction remains technically demanding, particularly when a transanal approach is chosen, and the low rectal anastomosis is especially prone to various complications [2–4]. Additionally, numerous errors have been reported in diagnosis and perioperative management [5]. The overall morbidity is considerable, with an early postoperative complication rate up to 18% [6] and a median of 3.5 surgical procedures required by five years of age, highlighting the complexity of care in this patient population [7]. Long-term sequelae, including faecal incontinence, obstructive defaecation and Hirschsprung-associated enterocolitis (HAEC) can impair quality of life well into adulthood [2].

Centralization policies have been widely adopted for various complex surgical conditions in adult surgery, supported by the well-established volume-outcome relationship, which demonstrates improved outcomes at high-volume centres [8–14]. In paediatric surgery, this relationship has been confirmed for conditions such as congenital diaphragmatic hernia repair, biliary atresia and in cardiac surgery [15–20]. Evidence for a similar relationship in HD surgery remains limited and inconclusive. Methodological challenges, including the rarity of HD, case heterogeneity, decentralized healthcare systems, and difficulties in associating long-term outcomes with the initial operating centre, complicate efforts to establish robust evidence.

The use of indirect evidence from common diseases for conditions where direct evidence is unattainable even under ideal study conditions has been recommended and is common practice for the guideline creation in rare diseases [21–24]. The rational for its use is especially clear for research questions, where no decision made due to the lack of direct evidence would be harmful to patients and thus the use of indirect evidence is necessary.

This study (1) presents a systematic review of the direct evidence on the volume–outcome relationship in HD corrective surgery and explores methodological challenges associated with such studies. (2) It assesses the feasibility of using indirect evidence to evaluate this relationship, applying the GRADE (Grading of Recommendations, Assessment, Development and Evaluation) framework [21], and synthesizes relevant findings. (3) Additionally, it examines the technical and perioperative similarities between adult colorectal surgery and HD corrective procedures.

## Methods

1. We performed a systematic review of English-language studies investigating the volume-outcome relationship in HD surgery. A comprehensive search of the MEDLINE database was performed for publications from January 1990 to December 2024 using the terms “Hirschsprung’s disease” AND (“volume outcome” OR caseload). The articles generated were processed and reported in accordance with the Preferred Reporting Items for Systematic reviews and Meta-Analyses (PRISMA) statement. Two independent reviewers (VS and MW) screened titles and abstracts for relevance. Full-text articles were retrieved for studies that met the inclusion criteria of investigating the volume-outcome relationship of HD. The full texts of selected articles were examined for data sources (e.g., registry, secondary data analysis), caseloads, outcome parameters, methods used to analyse the volume-outcome relationship, and the reported findings.
2. We reviewed the GRADE working group recommendations [21] for evaluating indirect evidence in rare diseases and examined the applicability for the evaluation of the volume-outcome relationship for HD corrective surgery:
3. The intervention differs from the intervention of interest, but the population and the outcome are the same
4. The outcome investigated may differ from the outcome of interest, but the population and the intervention are the same
5. The population investigated differs from the population of interest, but the intervention and the outcome are the same
6. We investigate commonalities between adult colorectal surgery and corrective surgery for HD. The core outcome parameters most frequently used in HD corrective surgery, as identified by Rossi et al. [25], were analysed regarding their underlying pathophysiology. To synthesize the extensive literature on volume-outcome relationship for adult colorectal surgery, we utilised the Cochrane review of Archampong et al. [26] and the V24-02 rapid report from the Institute for Quality and Efficiency in Health Care (Institut für Qualität und Wirtschaftlichkeit im Gesundheitswesen, IQWiG) [27]. Outcome measures from adult colorectal surgery were assessed for their applicability to paediatric HD surgery, considering anatomical similarities and comparable perioperative and postoperative challenges. Where available, caseload thresholds were also reported.

## Results

1. Systematic literature review of direct evidence on the volume-outcome relationship in HD corrective surgery

**Fig. 1:**
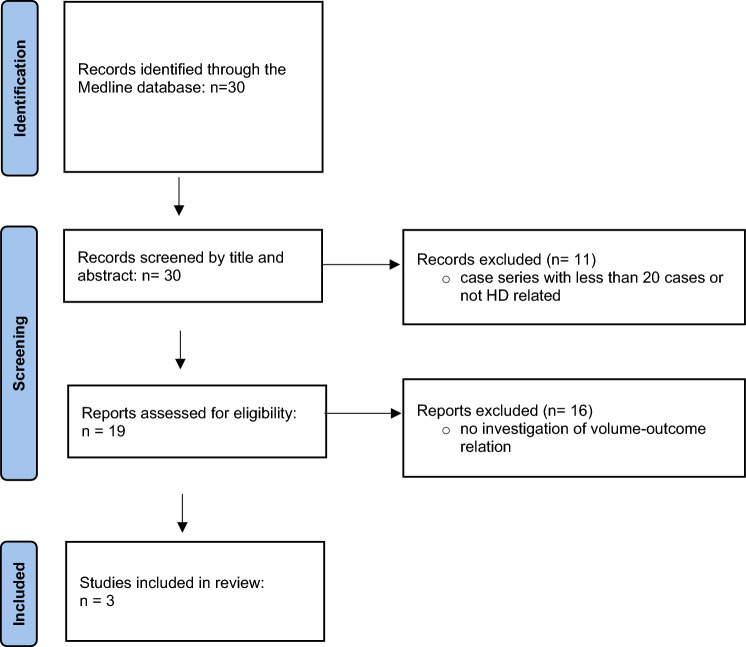
Prisma flow diagram showing selection of articles on volume-outcome in HD for review

The search strategy identified 30 studies from the MEDLINE database. Eleven studies were excluded by screening of title and abstract, as they either reported fewer than 20 cases, or did not focus on HD, or did not investigate a volume-outcome relationship (Fig. 1). The full-text articles of the remaining 19 studies were reviewed for the investigation of a volume-outcome relationship for HD. Of these, only 3 studies examined the relationship between surgical volume and outcomes (Fig. 1). All three included studies were retrospective, drawing data from either national health databases or single-institution records. Follow-up periods varied, with a maximum of two years (Table 1).

**Table 1:** Systematic literature review of direct evidence on the volume-outcome relationship in HD corrective surgery

	Apfeld et al. [28]	Söderström et al. [29]	Giuliani et al. [30]
Study design	retrospective analysis of hospital administrative data; multi-centre (41 centres)	retrospective analysis of hospital administrative data; single centre	retrospective analysis of hospital administrative data; multi-centre (21 centres)
Data source	Pediatric health information system (PHIS) database in the USA	Single-centre database of Stockholm University Hospital, Sweden	Hospital Episode Statistics (HES) database, UK
Follow-up time	2 years	unclear	1 year
Risk adjustment for the volume-outcome analysis (yes/no)	No	No	No
Case definition	Aggregated numbers of ostomy placement and corrective surgery	Corrective surgery	Aggregated numbers of ostomy placement, ostomy closure, biopsy, and corrective surgery
Definition of threshold	Surgeon volume: -low: < 2 procedures per year -high: > = 2 procedures per year Hospital volume: -low: < 10 procedures per year -high: > = 10 procedures per year	Hospital volume: -low: 7.2 corrective surgeries per year -high: 11.4 corrective surgeries per year	Hospital volume: -low: < 51 procedures per year -medium: 51–68 procedures per year -high: > 68 procedures per year
Outcomes			
Mortality	–	Low-volume group: 0.9% High-volume group: 0%	Overall: 1 year mortality: 2.8% (no significant difference)
Anastomotic leak/ intestinal perforation	–	Low-volume group: 8.3% High-volume group: 3.5%	–
Cumulative length of stay within 1st year of life	–	–	Overall: 40 days (no significant difference)
Reoperation	Overall: -within 30 days: 4.5% -within 2 years: 10.2%	Low-volume group: 12.3% High-volume group: 16.9%	Overall: 11.9% (no significant difference)
Re-admission	Overall: -within 30 days: 17.2% -within 2 years: 42.6%	Low-volume group: 15.8% High-volume group: 20.5%	Overall: 41% (no significant difference)
Volume-outcome relationship for the respective outcome parameter	No	No	No

– Not applicable

Apfeld et al. [28] used hospital discharge data from the Pediatric Health Information System (PHIS) to examine HD surgeries in 41 U.S. centres in patients under 60 days. Corrective surgery and stoma placement were used as aggregated numbers and not distinguished in the caseload definition or outcome analysis. Outcomes included reoperation and readmission within 30 days or two years. Surgeons and hospitals were stratified into volume groups (< 2 procedures vs. ≥ 2 procedures for surgeons;≤ 10 procedures vs. > 10 procedures for hospitals). Hospital caseloads were low (median (IQR) 9.5 (7–13) cases/year), limiting the statistical power to detect volume–outcome associations.

Söderström et al. [29] compared outcomes 5 years before and 5 years after national centralisation in 2018, which reduced the participating centres from 4 to 2 nationwide. Outcomes included hospital stay, HAEC, readmissions, reoperations and others. No significant differences were observed for reoperation rate, readmission, or intestinal perforation rates. The median annual caseload increased from 7.2 to 11.4 HD corrective surgeries per year, limiting the power to demonstrate a significant volume–outcome effect.

Giuliani et al. [30] analysed outcomes for procedures in HD patients across 21 hospitals in the United Kingdom using hospital discharge data (*n* = 1333). High-volume centres were defined as performing > 68 procedures/year, but the analysis aggregated numbers of diverse procedures, including corrective surgery, stoma formation, stoma closure and biopsies without stratification. The total number of corrective surgeries was 874 over a twelve-year period, resulting in 73 HD corrective surgeries per year on average. These were treated across 21 different hospitals, likely rendering the number of corrective surgeries in the “high-volume” centres well below 10 corrective surgeries per year. The authors state that “even high-volume hospitals in England had a small number of cases per year (5.6 for HD) and the average” probably referring to the number of corrective surgeries. Outcomes included 1-year mortality, reoperation, readmission, and cumulative length of stay. Key complications, such as anastomotic leaks, were not assessed. The authors found no significant association between hospital volume and outcomes.

Several challenges complicate the investigation of a volume–outcome relationship in corrective surgery for HD. Caseloads were generally low, making it difficult to establish distinctions between high and low-volume centres. Aggregating numbers of HD corrective surgeries with less complex “supportive procedures,” such as ostomy placement or closure, fails to account for the differences in surgical complexity and associated risks, making combined outcome analyses unreliable.

None of the available studies assessed long-term outcomes, such as faecal incontinence, quality of life, or patient satisfaction. This gap is particularly relevant in paediatric colorectal surgery, where mortality is low due to advances in modern healthcare, and long-term morbidity has become a primary concern.

The lack of risk adjustment represents a critical limitation. Approximately 30% of children with HD present with associated malformations, most notably congenital heart defects, which can significantly influence surgical outcomes [31–33].

(2) Methodological approaches when direct evidence for HD is unattainable: insight from the GRADE working group for the use of indirect evidence

The methodological challenges identified in the systematic review on the volume-outcome relationship for the surgical treatment of HD are not confined to HD but apply to a multitude of other rare conditions [22–24].

Methodological solutions have been developed for diseases where “direct evidence” is unattainable even under ideal study conditions. “Direct evidence” results from research that directly compares the interventions of interest (e.g. caseload of corrective surgery for HD per hospital) with the outcome of interest (e.g. mortality, morbidity) in the population of interest (e.g. neonates with HD). If either the population to which the evidence is applied differs or the intervention differs, or the outcomes differ, the evidence is “indirect”.

Three of the GRADE working groups categories of indirect evidence were examined for their applicability to the volume-outcome relation of corrective surgery for HD:

i. The intervention differs from the intervention of interest, but the population and the outcome are the same:

This type of evidence would ideally be drawn from more common colorectal conditions in the paediatric population other than HD. However, other conditions, such as anorectal malformations, are also rare and share the same challenges associated with rare disease research. However, for a multitude of complex paediatric surgical treatments, a clear volume-outcome relation was shown, such as surgery for congenital diaphragmatic hernia, biliary atresia and congenital heart defects [16–20]. The transferability of these studies to the volume-outcome relation for patients with HD can be based on the rational, that the complexity of these surgeries is comparable and thus the results of volume-outcome relations are transferable.

ii. The outcome investigated differs from the outcome of interest, but the population and the intervention are the same:

Volume-outcome studies often focus on mortality rates as an outcome parameter. However, mortality is not a useful outcome parameter for demonstrating a volume-outcome relation in paediatric colorectal surgery due to the very low overall mortality. McAteer et al. [15] provided a systematic review on volume-outcome relationships in paediatric surgery, including sixty-three studies on different complex malformations. For complex procedures where “mortality” or “failure of the original organ (e.g. liver)” is a significant outcome parameter, a volume-outcome relationship was evident, with a reduction in mortality observed at higher-volume hospitals. They noted that for most other complex paediatric procedures, in-hospital mortality is too rare to reliably measure the quality of care, and other primary outcome measures are more difficult to assess. Indirect evidence of this form can be used under the assumption that short-term outcomes that are easy to measure, such as the rate of minimal-invasive surgery can be a proxy for the quality of the operation and therefore for the long-term functional outcome. Wilms et al. found the rate of laparotomy in the decentralised HD surgical care in Germany with at least 40% to be higher compared to centralized settings.

iii. The population investigated differs from the population of interest, but the intervention and the outcome are the same:

From a surgical perspective, it seems feasible to examine the same procedure in a different population to identify the commonalities in technical surgical challenges and the learning curve for both the surgeon and the entire treatment team. This approach enables the transfer of expertise from a more prevalent population to a rarer one. While most adult colorectal surgeries are performed for malignancies, other indications include diverticulitis, rectal prolapse, and inflammatory bowel disease. Previous studies have shown that the technical demands of low rectal resection, such as nerve sparing dissection, preservation of the anal canal, and construction of a tension-free, well perfused anastomosis, are consistent regardless of the underlying pathology. Therefore, this type of indirect evidence is further explored in “Results”.

(3) Indirect evidence from the volume-outcome relationship from adult colorectal surgery

Using evidence from adult colorectal surgery to investigate the volume-outcome relationship in paediatric corrective surgery represents a form of indirect evidence, where the population investigated differs from the population of interest, but the intervention and outcome measures are comparable. In our study, the intervention under consideration is the low rectal resection, extending orally to various levels. The comparable complexity of this surgical intervention is examined in detail under the section “Considerations of Surgical Anatomy and Key Technical Similarities”, while the relevance of outcomes measures is addressed in “Considerations of Core Outcome Parameters”. Given the abundance of literature for the volume-outcome relationship in adult colorectal surgery, we refer mainly to the systematic literature review by Archampong et al. [26] and the IQWiG v024-02 report [27], report as foundational sources for summarizing current evidence.

## Considerations of surgical anatomy and key technical similarities

The most common presentation of HD is the rectosigmoid aganglionosis that consistently affects at least a portion of the rectum. However, HD the extend of aganglionosis can vary, with the most severe cases involving the whole colon and even the small bowel. The principal surgical techniques for corrective surgery include either a proctectomy [34], a rectal mucosectomy [35], or a bypass procedure with preservation of parts of the rectum [36]. The proximal resection margin is determined by the extent of the aganglionosis, resulting in a range of resections from isolated rectal or rectosigmoidal resections, to left colectomies with rectal resection, extended left colectomies, or total colectomy. In cases where the aganglionosis extends to the proximal transverse colon, surgical manoeuvres such as a Deloyers or Turnbull procedure might be necessary [37]. The surgical approach can be either transanal [38] and/or abdominal via laparotomy or minimally invasive [39].

In adult colorectal surgery, most colorectal procedures are for the treatment of colorectal cancer. In case of rectal cancer total mesorectal excision (TME) is performed with a low rectal anastomosis. Like most adult colorectal patients, paediatric patients with HD generally have an intact sphincter mechanism and anal canal, providing the anatomical prerequisites for faecal continence. In both populations, the primary goals are to achieve curative resection margins while minimizing post-surgical functional disorders. This includes preserving the sphincter mechanism, anal canal and avoiding injury to adjacent structures. A key technical commonality between adult and paediatric colorectal surgeries is the performance of a low, tension-free rectal anastomosis, which carries similar risks of complications such as anastomotic failure and stenosis. Other common pitfalls in colorectal surgery include dissection in the wrong anatomical plane, which can lead to injury of surrounding organs and neurovascular structures. The challenges associated with the “holy plane” dissection in TME, aimed at carefully removing the rectum and mesorectum *en bloc* while preserving the mesorectal fascia, which is critical for achieving an oncological complete resection, are technically comparable to the complexity of rectal pull-through dissection performed in HD [40]. Both procedures require meticulous technique to preserve surrounding structures and ensure optimal functional outcomes. [3–5, 40]. Despite differences in underlying pathologies, colorectal surgery in adult and paediatric patients share key anatomical and technical similarities.

## Considerations of similarities of core outcome parameters

In the systematic literature review of Rossi et al. [25], a total of 200 studies were identified, measuring 601 different outcome parameters. Of these, 116 outcomes were reported in at least 5% of the publications. The 10 most frequently investigated outcome were: postoperative HAEC (63.5%), faecal incontinence (53.5%), constipation (48%), anastomotic stricture (38%), anastomotic leakage (28%), length of initial in-hospital stay (35.5%), mortality (26.5%), reoperation rate (42.5%), wound infection (14%) and hospital readmission rate (12.5%).

In this study, we explore these 10 outcome parameters with respect to their pathogenesis and the associated technical challenges involved in their prevention. We then assess whether these outcomes are also considered relevant in the literature on adult rectal surgery and, where applicable, whether a volume-outcome relationship exists for this parameter. HAEC, being specific to HD and not directly reflective of surgical technique or intraoperative quality, was excluded from further analysis.

To synthesize existing evidence on the volume-outcome relationship in adult colorectal surgery, we relied on two major sources: the IQWiG rapid report v24-02 [27] and the systematic review by Archampong et al. [26] were used. The IQWiG rapid report v24-02 included 14 studies on rectal surgery, of which 7 also evaluated colon surgery. The systematic review of Archampong et al. [26] encompasses 51 studies. Both sources provided detailed analyses of hospital- and surgeons-level volume-outcome associations. The results from those sub-analysis, including rectal resections, are shown in Table 2.

**Table 2:** Indirect evidence from the volume-outcome relationship from adult colorectal surgery

	Archampong et al. [26] (unadjusted)		IQWiG report [27]	
	Surgeon VO (yes/no)	Hospital VO (yes/no)	Surgeon VO (yes/no)	Hospital VO (yes/no)
Faecal incontinence	–	–	–	–
Permanent stoma rate as indirect parameter for anastomotic problems	Yes: 0.75 [0.62, 0.89]*	Yes: 0.86 [0.75, 1.00]*	Yes: 1 out of 1 study	Yes: 2 out of 3 studies**
Constipation/obstructive defecation	–	–	–	–
Anastomotic stricture	–	–	–	–
Anastomotic leak	Yes: 0.67 [0.49, 0.92]*	No: 1.18 [0.87, 1.58]*	–	–
Length of primary hospital stay	–	–	Yes: 1 out of 1 study	Yes: 1 out of 3 studies**
Early Mortality (within hospital stay or 30-days postoperatively)	Yes: 0.65 [0.57, 0.75]*	Yes: 0.75 [0.68, 0.84]*	Yes: 1 out of 1 study	yes: 5 out of 9 studies**
Reoperation Rate	–	–	No: 1 out of 1 study**	No: 1 out of 1 study**
Wound infection	–	–	–	no: 1 out of 1 study
Readmission Rate	–	–	No: 1 out of 1 study**	No: 2 out of 2 studies**

– Not evaluated

*VO* volume-outcome

*odds ratio with CI 95% in favour of the respective high-volume category, **those study that did not reveal significant results with better outcomes in high-volume hospitals also did not reveal significant results with better outcomes in low-volume hospitals

### Faecal incontinence

The systematic review of Dai et al. [41] on long-term outcomes of HD reported a pooled incontinence rate of 0.20 (95% CI 0.13–0.28) highlighting its significance as an outcome parameter. Neither in the systematic review from Archampong et al. [26] nor in the IQWiG report [27] faecal incontinence was considered as an outcome parameter. Possibly, this is due to its subjectivity in nuances and the possible undercoding, especially when hospital discharge data is used. However, “permanent stoma rate” defined as the existence of a stoma longer than 12 months postoperatively was used as a proxy outcome parameter.

In Archampong et al. [26] higher hospital volume and higher surgeon volume were also associated with significantly lower rates of permanent stomas 0.86 (95% CI 0.75–1.00) and 0.75 (95% CI 0.62 to 0.89), respectively. The IQWiG report [26] equally found a volume-outcome relationship for the outcome parameter “permanent stoma rate” based on the study of Aquina et al. [42] on the hospital level with OR 0.93 (95% CI 0.91–0.96) for an increase of 10 cases per hospital and year in a continuous analysis. It also found a volume-outcome relation for surgeon volume with an OR 0,85 (CI 0.78; 0.94) for an increase of 10 cases per surgeon and year in a continuous analysis.

### Constipation/obstructive defecation syndrome

The systematic review of Dai et al. [41] on long-term outcomes of HD reported a pooled rate of constipation or obstructive defecation syndrome of 0.14 (95% CI 0.06–0.25), making it a relevant outcome parameter. In contrast, this outcome parameter was not included in the systematic review from Archampong et al. [26] or in the IQWiG report [27], possibly due to its subjective nature and possible underreporting, especially when based on hospital discharge data. Nevertheless, constipation and obstructive defecation syndrome are well-recognised long-term complications, and the incidence of low anterior resection syndrome (LARS) was assessed at 44% (95% CI 40–48%; *I*^2^ = 88%; 36 studies) in the systematic review by Sun et al. [43]. With anastomotic leak rate being an independent risk factor for LARS, a volume-outcome relationship is likely.

Anastomotic stricture: Neither Archampong et al. [26] nor the IQWiG report [27], included “anastomotic stricture” as an outcome parameter. This may be due to the nuanced and subjective nature of the diagnosis, as well as the possibility of underreporting, especially when hospital discharge data is used. These cases would be measurable either under “permanent stoma rate” or “reoperation” as a relevant stenosis would prevent the normal intestinal passage. The volume-outcome relation for “permanent stoma rate” can apply to this adverse outcome.

Anastomotic leak: In the comprehensive national dataset of all 1,166 corrective surgeries for HD performed in Germany between 2016 and 2022, Wilms et al. [6] found an anastomotic leak rate of 3.6% and a rate of peritonitis/intestinal perforation of 6.6%.

In the systematic reviews by Archampong et al. [26], the anastomotic leak rates were significantly lower among high-volume surgeons. The difference in the incidence of anastomotic leaks between low and high-volume hospitals was not statistically significant. The IQWiG report did not analyse anastomotic leak rates as an outcome parameter [27].

Length of primary hospital stay: In the dataset of corrective surgeries for HD performed in Germany, Wilms et al. [6] reported a median length of hospital stay of 10 days (IQR 7–15 days). Archampong et al. [26] did not analyse the volume-outcome relationship for the length of primary hospital stay. The IQWiG report [27] did not find a significant volume-outcome relationship for length of stay in the subgroup of rectal resections. For the broader group of colorectal resections, both higher-volume hospitals and higher-volume surgeons were associated with significantly shorter hospital stays. Burns et al. [44] examined 109,261 cases of colorectal resection in adults and observed a significantly shorter hospital stay in hospitals performing 69 or more colorectal resections per year. Similarly, Pucciarelli et al. [45] in an analysis of 353,941 cases, found that a higher hospital caseload was associated with shorter length of stay, with a statistically significant difference in hospitals performing at least 44 colorectal resections per year.

Mortality: In the national dataset of Wilms et al. [6], the in-hospital mortality for HD was zero. Mortality is not a relevant outcome parameter in children with HD; rather morbidity, particularly within the first 5 years of life, is significantly more prevalent [7]. Archampong et al. [26] as well as the IQWiG report found that, with respect to the outcome parameter of short-term mortality, defined as a 30-day mortality or in-hospital death, there was an observed association between higher hospital-volume as well as surgeon-volume and improved treatment quality.

Reoperation rate: The outcome measure of unplanned reintervention within 30 days showed no association with either hospital or surgeon volume [26]. This association was not analysed at the combined hospital and physician case volume level. The study by Archampong et al. [26] did not include this outcome parameter (Table 2). This limited reporting may be attributed to the challenges of capturing such data, particularly in retrospective analyses.

Wound infection: In the IQWiG report there was no association between hospital volume and treatment quality with respect to postoperative wound infections. This association was not examined at the physician level or at the combined hospital and physician case volume level. This outcome parameter was not analysed by Archampong et al. [26] (Table 2).

Readmission rate: The 30-day readmission rate following corrective surgery was poorly studied. In the IQWiG report, based on two studies with inconclusive findings, no association was found between hospital volume and the outcome parameter of hospital readmission. Similarly, at the surgeon level, one study with inconclusive results showed no association between surgeon volume for this outcome. No analysis was conducted at the combined hospital and surgeon volume level [26].

## Discussion

Successful surgical correction and follow-up care remain central to achieving optimal outcomes in HD [2–5, 38, 41]. A positive correlation between surgical volume and patient outcomes in adult surgery has been well established [47], with numerous studies, including in colorectal procedures, consistently confirming this volume–outcome relationship [26, 44–46, 48].

This systematic literature review on the volume–outcome relationship in HD corrective surgery identified three studies. All studies were limited by overall low hospital and surgeon caseloads. Notably, the “high-volume” category in each study included centres performing fewer than 12 corrective HD surgeries per year. Furthermore, long-term functional outcomes were not assessed, and risk stratification was unfeasible due to the small sample sizes. These methodological limitations reflect the inherent challenges due to the rarity of HD and the fragmentation of care in decentralized healthcare systems. As such, they cannot be overcome solely through improved study designs.

Extremely low case volumes not only hinder volume–outcome research but also limit the potential for robust clinical research in general. Therefore, centralization of care is essential to improve both the quality of treatment and the strength of future studies relying on direct evidence, as statistically significant results require higher patient numbers [46].

Evidence for a volume-outcome relationship could previously be derived from the comparison of outcomes of HD corrective surgery between decentralized health care systems and high-volume single centres. The comparison between early postoperative complication rates of Germany’s decentralised health care structure in the study of Wilms et al. [6] with the results of the high-volume single centre study by Zhang et al. [49] showed a relevant difference in the need for a laparotomy as compared to minimal-invasive techniques, the hospital length of stay, the anastomotic leak rate and the revision rate.

For rare conditions such as HD, expert opinion plays a vital role in guideline development due to limited high-level evidence. The ERNICA guideline for the treatment of HD is based on all 33 of its statements across nine key areas on expert consensus, with only a few supported by empirical evidence [39]. Several statements emphasize the importance of specialized centres and multidisciplinary care. The panel unanimously agreed that “concentration of interdisciplinary experience is associated with better outcomes in complex or rare paediatric surgical conditions” and that “the need for re-do surgery is low in centres that regularly manage HD, and complications are appropriately identified and managed.” Furthermore, patient organizations, recognized as “experts by experience”, have long advocated for centralization, citing ongoing concerns about the quality of care in low-volume centres [6].

The three types of indirect evidence recommended by the GRADE working group for rare diseases are applicable to investigating the volume-outcome relationship in corrective surgery for HD. Indirect evidence from other complex paediatric procedures, such as for biliary atresia and congenital diaphragmatic hernia, is feasible, especially in demonstrating the complexity of perioperative management. When focusing on the technical surgical challenges, examining the same procedure and the same outcome in a different population is preferrable.

An extensive body of research supports a strong volume–outcome relationship in adult colorectal surgery. Both original studies and systematic reviews consistently demonstrate that higher hospital and, in some cases, surgeon volumes correlate with improved outcomes. While most studies focus on colorectal cancer, similar findings have been reported in surgeries for diverticulitis and rectal perforation [8]. These results suggest that the complexity of colorectal surgery is primarily anatomical rather than diagnosis-specific, supporting extrapolation to paediatric HD surgery. Systematic reviews using continuous caseload analyses have identified significant outcome improvements above certain volume thresholds. In most, a caseload below 12 colorectal resections per year failed to show a significant volume–outcome relationship. This finding is particularly concerning, as many paediatric surgical units continue to perform fewer than 12 HD corrective procedures annually, limiting their ability to optimize outcomes.

## Conclusion

Due to the rarity of HD and low procedural volumes, a volume–outcome relationship in HD corrective surgery cannot currently be confirmed or refuted using direct evidence. These limitations are inherent and cannot be resolved through improved study design alone. In such cases, indirect evidence is both necessary and appropriate. The well-established volume–outcome relationship in adult colorectal surgery, independent of diagnosis, strongly supports the existence of a similar relationship in HD. The shared complexity of rectal resections and adverse event profiles across age groups further justifies this extrapolation. Given that improved outcomes in adult colorectal surgery are observed above a threshold of 12 cases per year, the low procedural volumes in paediatric units suggest considerable room for quality improvement. Centralization of care should be prioritized to enhance outcomes and enable stronger evidence generation in HD corrective surgery.

## Data Availability

No datasets were generated or analysed during the current study.
